# Females Fly Further—Sex‐Specific and Seasonal Differences in Migration Patterns of Pied Avocets

**DOI:** 10.1002/ece3.73798

**Published:** 2026-06-11

**Authors:** Mads Eskildsen, Stefan Garthe, Sjoerd Duijns, Petra Manche, Dominik Marchowski, Łukasz Jankowiak, Marcin Sidelnik, Philipp Schwemmer

**Affiliations:** ^1^ Research and Technology Centre (FTZ) Kiel University Büsum Germany; ^2^ Sovon Dutch Centre for Field Ornithology Nijmegen the Netherlands; ^3^ Ornithological Station, Museum and Institute of Zoology Polish Academy of Sciences Gdańsk Poland; ^4^ Department of Ecology and Anthropology, Institute of Biology University of Szczecin Szczecin Poland; ^5^ Nature Conservation Foundation “Na Skrzydłach” Warszawa Poland

**Keywords:** annual cycle, GPS tracking, migration distance, migration timing, shorebird

## Abstract

Migration patterns in birds are shaped by ecological and physiological constraints that differ between sexes, seasons and among breeding regions, resulting in complex temporal and spatial strategies. Understanding this variation is crucial for revealing how individuals optimise their annual cycles. As very little is known about the individual migratory behaviour of pied avocets ( *Recurvirostra avosetta* ), this study investigated how spatiotemporal migration patterns of avocets breeding in northern Europe vary seasonally, sexually, and spatially. We captured 122 adults on their nests in three countries using walk‐in traps and equipped them with lightweight (6–9 g) solar‐powered GPS loggers. Avocets spent an extended post‐breeding period (on average until early November) in the Wadden Sea before commencing autumn migration. While most individuals followed the East Atlantic Flyway, four deviated via an inland route across the Alps to Italian wintering sites. On average, avocets travelled 1503 ± 996 km within 13.3 ± 19.8 days, resulting in a mean migration speed of 470.8 ± 554.0 km day^−1^. The main wintering range was concentrated in France and Portugal, with the Tejo Estuary being the most frequently used site. Migration tactics differed between seasons and sexes: autumn migration lasted longer and involved fewer but extended stopovers, whereas spring migration was faster, with more but shorter stopovers. Females migrated significantly further (1928 km vs. 1277 km in males) and wintered at more southern latitudes, while males remained longer in the Wadden Sea in autumn and tended to arrive at breeding sites earlier in spring. Avocets breeding in the Wadden Sea tended to winter further south than those from the Baltic Sea (Poland), most of which first moved to the Wadden Sea before continuing migration. Our findings highlight the central role of the Wadden Sea as a key fuelling area in the annual cycle of pied avocets from northern Europe.

## Graphical abstract

GPS tracking of 122 pied avocets revealed pronounced sex‐ and season‐specific migration strategies, with females migrating further than males, while spring migration was faster and more fragmented than autumn migration. Avocets spent a substantial part of their annual cycle in the Wadden Sea during the post‐breeding period before starting migration to their wintering sites in western Europe and beyond. These patterns highlight how sex‐specific strategies and seasonal constraints jointly shape the annual cycle of this coastal wader.

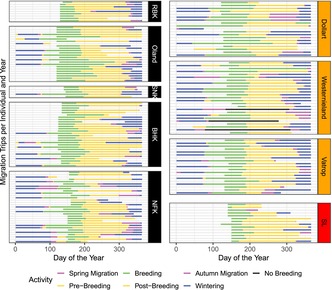

## 1 Introduction

Pied avocets ( *Recurvirostra avosetta* ; hereafter avocets) have experienced significant population declines in the Wadden Sea in recent decades (Kleefstra et al. 2025; Koffijberg et al. 2026). Although the Wadden Sea represents a central hub for migratory and breeding avocets of the East Atlantic Flyway (EAF) population, the proportion of individuals using the area has also declined markedly, from 53% between 1999 and 2007 (Laursen et al. 2010) to 25% between 2013 and 2023 (Kleefstra et al. 2025). The pied avocet is listed in Annex I of the EU Birds Directive and thus is subject to special conservation measures (European Parliament 2019). Despite this conservation relevance, the individual migratory patterns of avocets along the EAF remain poorly understood. However, the development of effective conservation strategies requires detailed knowledge of the ecological basis of species' annual cycles. In this study, we aim to fill these knowledge gaps by describing the migration ecology of avocets.

Migration is an essential component of the annual cycle of migratory bird species, linking breeding and wintering sites, and its timing can be crucial for breeding success (Hötker 1998; Newton 2008; Rappole 2013; Schmaljohann et al. 2022). Shifts in migration timing towards later onsets of both spring and autumn migrations have been observed in several species in the past decade (Mondain‐Monval et al. 2021), probably as a consequence of changing environmental conditions.

Timing of departure from the breeding site is strongly influenced by parental roles; if one sex ceases parental care earlier, it may also depart earlier (Bauer et al. 2005; Newton 2011; Pederson et al. 2022; Wright et al. 2022; Ersoy et al. 2024), while species in which both sexes share chick rearing often show no difference in departure timing between males and females (Bauer et al. 2005; Newton 2011; Baert et al. 2018). Notably, however, males of many species arrive earlier at breeding sites in spring to secure high‐quality territories and maximise their mating opportunities (Alerstam 2011; Newton 2011; Schmaljohann et al. 2022).

Many wader species display sexual size dimorphism (Jehl and Murray 1986; Bauer et al. 2005), and sex‐specific differences in migration patterns may thus also arise from underlying physiological traits. Wing loading (ratio of body mass to wing area) quantifies the relative wing size and reflects flight efficiency (Vágási et al. 2016). A lower wing loading increases the bird's flight economy and long‐distance flight performance (Norberg 1990; Vágási et al. 2016), potentially allowing individuals with a lower wing loading to migrate further.

Seasonal differences between spring and autumn migrations are well documented. Most birds migrate faster in spring, when selection pressures favour their rapid arrival at breeding sites, whereas autumn migration is typically slower and includes longer stopovers (Nilsson et al. 2013; Schmaljohann et al. 2022). In spring, birds may have accumulated larger fuel reserves before departure and are under strong time constraints due to competition for territories, resulting in shorter, more efficient stopovers (Nilsson et al. 2013; Schmaljohann 2018). Waders and gulls in particular tend to show faster migration speeds during spring migration (Klaassen et al. 2012; Schmaljohann 2018; Duijns et al. 2019; Wright et al. 2022; Catry et al. 2024); however, exceptions exist, such as Mediterranean gulls ( *Ichthyaetus melanocephalus* ), which migrate faster in autumn than in spring (Jankowiak et al. 2024), and a similar pattern has been reported for Icelandic whimbrels ( *Numenius phaeopus islandicus* ) (Carneiro et al. 2019).

Avocets of the EAF population winter between The Netherlands and the United Kingdom in the north and South Africa in the south (Agblonon et al. 2023). Although the core wintering range is located between France and Morocco, numbers in the United Kingdom have increased markedly over recent decades (Rehfisch et al. 2003; van Roomen et al. 2020, 2025; Agblonon et al. 2023), indicating a trend towards shorter migration distances, likely driven by climate change (Visser et al. 2009; Lok et al. 2013). Avocets breeding in France show partial migration, with most individuals (86%) remaining resident, and of the 24% that are migratory, 81% winter on the Iberian Peninsula (Chambon et al. 2018).

The timing of autumn migration in avocets is strongly temperature‐dependent, resembling a stepwise ‘escape from the cold’ (Glutz von Blotzheim et al. 1986). This behaviour can lead to pronounced interannual variation in migration patterns, depending on winter severity. Both pied and American avocets ( *Recurvirostra americana* ) exhibit higher intraspecific variability in migration patterns than most other waders (Clements et al. 2025; Eskildsen et al. 2025). Their comparatively short migration distances allow greater behavioural flexibility, whereas species migrating over longer distances are more constrained in terms of their timing and route choice (Pederson et al. 2022; Catry et al. 2024).

In this study, we analysed the temporal and spatial migration patterns of avocets breeding in the German and Dutch Wadden Sea and in Poland (Baltic Sea), with the aim of characterising the phenology of the annual cycle and identifying key sites used throughout the year. Specifically, we examined differences in wintering latitude, number of stopovers, migration distance, speed, and duration, as well as migration timing in relation to sex, season (spring vs. autumn), and breeding region (Wadden Sea vs. Baltic Sea).

We made the following five predictions about avocet migration patterns: (I) avocets exhibit short migration distances and a late onset of migration, reflecting a prolonged post‐breeding period (Pederson et al. 2022; Catry et al. 2024; Eskildsen et al. 2025); (II) females have been reported to winter further south than males (Hötker 1998; Dietrich 1999) and we therefore expected them to show lower wintering latitudes, longer migration distances and durations, and a lower relative wing loading index, and to depart earlier from the Wadden Sea; (III) male breeding success decreases with later arrival (Hötker 1998) and males are assumed to winter closer to the breeding sites, and we therefore predicted that males would arrive earlier than females; (IV) we expected shorter migration durations, fewer stopovers, and higher migration speeds in spring than in autumn, in accordance with other species (Nilsson et al. 2013; Schmaljohann 2018; Schmaljohann et al. 2022); and (V) some avocets from the Polish breeding colony move to the Wadden Sea during the post‐breeding period (Marchowski et al. 2023), and we thus predicted that Wadden Sea breeders might represent a further segment in a chain‐migration system, and might therefore winter further south and undertake longer migrations than Baltic Sea breeders.

## 2 Materials and Methods

### 2.1 Tagging of Avocets

Avocets were caught on their nests during incubation using walk‐in traps. Between 2020 and 2024, a total of 122 adult avocets were equipped with Global System for Mobile Communication (GSM) Global Positioning System (GPS) dataloggers. Nine individuals were excluded from the analyses due to tag loss or malfunction (*n* = 6) or mortality (*n* = 3) within the first few days to weeks after capture, resulting in 113 individuals used in this study. Of these, 70 individuals were captured in the German Wadden Sea (Beltringharder Koog (*n* = 22; 54°32′29″ N, 8°54′17″ E), Neufelderkoog (*n* = 18; 53°53′29″ N, 8°58′25″ E), Oland (*n* = 17; 54°40′44″ N, 8°42′18″ E), Rickelsbüller Koog (*n* = 9; 54°53′08″ N, 8°38′59″ E), Sönke‐Nissen‐Koog (*n* = 4; 54°36′33″ N, 8°51′35″ E)), 29 in the Dutch Wadden Sea (Westernieland (*n* = 11; 53°25′28″ N, 6°31′21″ E), Vatrop (*n* = 9; 52°56′04″ N, 5°00′16″ E), Dollart (*n* = 9; 53°17′32″ N, 7°09′33″ E)), and 14 in the Szczecin Lagoon (Poland; 53°47′33″ N, 14°27′07″ E).

All birds were weighed to the nearest gram, measured (tarsus length, wing length (carpal joint to longest primary), bill length) to the nearest mm, and ringed (colour ring, metal ring). Three different types of solar‐powered tags from two manufacturers were used, weighing 1.8%–2.7% of the tagged bird's mean body mass (332 ± 27 g). This remained below the generally accepted threshold of 3% of body mass to avoid behavioural alterations caused by tagging (Barron et al. 2010; Geen et al. 2019). Two tag models were produced by Ornitela, Lithuania (OT‐6 (6 g, *n* = 29), OT‐9 (9 g, *n* = 70)) and one model by Interrex, Poland (INTERREX 18 (8.9 g, *n* = 14)). The loggers were attached to the bird's back using either a body harness (*n* = 84) or a leg‐loop harness (*n* = 29) (Thaxter et al. 2014). Fix intervals ranged from one location per minute to one per day according to the energy levels of the devices (mean: 138.4 ± 107.6 GPS fixes per day). A total of 81 individuals were sexed genetically (Tauros Diagnostik GbR, Bielefeld, Germany; Institute of Avian Research, Wilhelmshaven, Germany), identifying 45 males and 36 females, while the sexes of the other 32 individuals remained unknown (Table 1).

**TABLE 1:** Overview of tagging information.

	Germany	The Netherlands	Poland	Total
Tagged birds	70	29	14	113
Males	40	5	—	45
Females	30	6	—	36
Unsexed	—	18	14	32
*n*	57	26	8	91
Spring migration	27	35	—	62
Autumn migration	70 (+ 20)	58 (+ 4)	8 (+ 6)	136 (+ 30)
Device type	OT‐6, OT‐9	OT‐9	INTERREX 18	
Study period	2020–2024	2021–2024	2023–2024	2020–2024
Attachment method	Body harness	Leg‐loop harness	Body harness	

*Note:* Numbers in brackets indicate incomplete autumn migration trips. *n* = number of individuals that completed at least one migration trip. For detailed information about each individual see Table A1.

### 2.2 Migratory Variables

Ninety‐one (81%) of the tagged individuals performed at least one complete migration trip (i.e., complete trip from the breeding to the wintering location or vice versa), of which 38% (*n* = 35) also had recorded data for consecutive years, up to a maximum of 4 years. A total of 228 migration tracks were recorded, comprising 136 complete autumn migration tracks (plus 30 incomplete tracks) and 62 complete spring migration tracks. For three of the incomplete autumn tracks, the signal was lost at a possible wintering site, i.e., a site where other avocets wintered, between 20 October and 2 January; however, onward migration to a different site could not be definitely ruled out, and these tracks were therefore classified as incomplete. A migration track was defined as complete if the individual had left the Wadden Sea and subsequently stayed at a known wintering site for > 14 days, based on the mean stopover duration of 13.4 days reported for avocets (Eskildsen et al. 2025). When considering only stopovers made after departure from the Wadden Sea, the mean stopover duration decreases to 4.9 days, and only 7% (4 out of 55) of stopovers exceeded 14 days (Eskildsen et al. 2025). Consequently, a stay of more than 14 days following migration is highly likely to represent a wintering site.

Breeding and wintering sites were identified for each individual. Breeding sites corresponded to the respective nesting sites, which were determined either through direct field observations in the year of capture or inferred visually from GPS data in subsequent years, indicated by nest‐typical movements (i.e., small‐scale point clusters with frequent arrivals and departures). Stopovers were defined as interruptions of migratory flight lasting > 1 h at a single site, following Pederson et al. (2022) and Eskildsen et al. (2025). Because some devices did not maintain sub‐hourly fix intervals, not every stopover event may have been detected, potentially resulting in a slight underestimation of the number of stopovers. Departure from and arrival at breeding sites were identified as the last GPS fix at the breeding/wintering site before migration commenced, and the first fix at the wintering/breeding site upon arrival, respectively. In cases where individuals used multiple breeding or wintering sites within the same year, the first site was used to determine arrival, and the last site for departure.

Given that avocets spend extended periods in the Wadden Sea during their post‐breeding phases (Eskildsen et al. 2025), departure from and arrival at the Wadden Sea were additionally recorded to describe migration timing. Post‐ and pre‐breeding periods therefore represent the time between departure from the breeding site and departure from the Wadden Sea, and the time between arrival at the Wadden Sea and arrival at the breeding site, respectively. Autumn migration duration was calculated as the time between departure from the Wadden Sea and arrival at the wintering site, and spring migration as the time between departure from the wintering site and arrival at the Wadden Sea. Migration distance between the breeding and wintering sites was calculated as the great circle distance using Vincenty's formulae in the R package geosphere (Vincenty 1975; Hijmans 2022). This calculation therefore does not account for the actual migratory path travelled by the birds. The number of stopovers (between the Wadden Sea and wintering site and vice versa), migration duration, and migration distance were determined for each complete migration trip. Additionally, the migration speed was calculated as the quotient of migration distance and migration duration, without considering pre‐migratory fuelling (Alerstam 2003). Some of these variables could not be calculated for every migration track because of minor data gaps, resulting in variable sample sizes across migratory variables. The sample sizes are therefore indicated in the relevant figure captions.

### 2.3 Statistical Analysis

We investigated the seasonal, regional, and sex‐specific differences in migration patterns using a series of generalised linear mixed models (GLMMs) using the R packages lme4 (Bates et al. 2015) and glmmTMB (Brooks et al. 2017). Bird ID was included as a random effect in all models to account for repeated measurements of the same individuals. We first tested the effect of season (spring vs. autumn migration) on the number of stopovers, migration duration, and migration speed. We then examined the influence of the breeding region (Wadden Sea vs. Baltic Sea) on wintering latitude, migration distance, departure from breeding site, departure from Wadden Sea, arrival at wintering site, number of stopovers, migration duration, and migration speed. Because no spring migration trips were recorded for the Baltic Sea breeders (Table 1), only autumn migration parameters were included in this comparison. Third, we assessed sex‐specific differences (male vs. female) in wintering latitude, migration distance, departure from breeding site, departure from Wadden Sea, arrival at wintering site, departure from wintering site, arrival at the Wadden Sea, and arrival at the breeding site. We finally analysed the combined effect of sex and season, including their interaction, on the number of stopovers, migration duration, and migration speed.

The most appropriate probability distribution for each response variable was selected based on the Akaike information criterion (Akaike 1998). Two different distributions were applied for some response variables (number of stopovers, migration duration, wintering latitude, departure from Wadden Sea, and arrival at wintering site) due to varying sample sizes across predictor variables (Table 2). The significance of the fixed effects (season, region and sex) in the GLMMs was assessed using likelihood‐ratio tests (LRTs) by comparing full models with corresponding null models lacking the respective fixed effect. For the combined effect of sex and season, we subsequently conducted pairwise post hoc comparisons using estimated marginal means (EMMs) from the R package emmeans (Lenth and Piaskowski 2025).

**TABLE 2:** Input data and results of the generalised linear mixed models (GLMM).

Model	IndivNo	TripNo	*n*	ProbDistr	*t*/*z*‐value	*p*
Autumn & spring migration						
StopoverNo~Sex*Season	81	159	122	Poisson	See Table A2	
StopoverNo~Season	113	228	172	Tweedie	*z* = 1.90	< 0.001***
StopoverNo~Region	113	166	115	Poisson	*z* = 2.22	0.015*
MigrationDur~Sex*Season	81	159	135	Neg. binomial	See Table A3	
MigrationDur~Season	113	228	189	Tweedie	*z* = −3.16	0.002**
MigrationDur~Region	113	166	130	Tweedie	*z* = 2.57	0.014*
MigrationSpeed~Sex*Season	81	159	131	Gamma	See Table A4	
MigrationSpeed~Season	113	228	180	Gamma	*z* = 2.76	0.005**
MigrationSpeed~Region	113	166	128	Gamma	*z* = −3.46	< 0.001***
Autumn migration						
WinteringLat~Region	113	166	136	Gaussian	*t* = −1.96	0.050*
WinteringLat~Sex	81	116	95	Tweedie	*z* = 2.51	0.019*
MigrationDist~Region	113	166	132	Tweedie	*z* = 1.60	0.112n.s.
MigrationDist~Sex	81	116	94	Tweedie	*z* = −3.08	< 0.001***
DepBreeding~Region	113	166	162	Gamma	*z* = 0.77	0.440n.s.
DepBreeding~Sex	81	116	115	Gamma	*z* = −0.40	0.707n.s.
DepWadden~Region	113	166	144	Gaussian	*t* = −2.03	0.043*
DepWadden~Sex	81	116	101	Tweedie	*z* = 4.24	< 0.001***
ArrWintering~Region	113	166	131	Gaussian	*t* = −1.66	0.095n.s.
ArrWintering~Sex	81	116	93	Tweedie	*z* = 3.24	0.008**
Spring migration						
DepWintering~Sex	34	43	42	Tweedie	*z* = 0.51	< 0.001***
ArrWadden~Sex	34	43	40	Gamma	*z* = −0.80	0.426n.s.
ArrBreeding~Sex	34	43	39	Gamma	*z* = −1.43	0.161n.s.

*Note:* Significance levels: n.s. not significant, *p < 0.05, **p < 0.01, ***p < 0.001.

Abbreviations: ArrBreeding, arrival at breeding site; ArrWadden, arrival at Wadden Sea; ArrWintering, arrival at wintering site; DepBreeding, departure from breeding site; DepWadden, departure from Wadden Sea; DepWintering, departure from wintering site; IndivNo, number of individuals in the dataset; MigrationDist, migration distance; MigrationDur, migration duration; *n*, number of trips used from the dataset (i.e., for the model in the first row 159 trips were in the dataset but only 122 of them contained information about the number of stopovers); ProbDistr, probability distribution used for the respective GLMM; Region, breeding region (Wadden Sea vs. Baltic Sea); StopoverNo, number of stopovers; TripNo, number of trips in the dataset; WinteringLat, wintering latitude.

In addition, we determined if migration distance was associated with the timing of departure from the breeding site using Spearman's rank correlation. As wing area was not recorded during field work and was therefore unavailable for calculating wing loading, we calculated a relative wing loading index as body mass divided by wing length for individuals with available measurements (*n* = 92). Viscor and Fuster (1987) reported strong correlations between wing area and wing length based on a dataset of 346 bird species, including avocets. We therefore considered the relative wing loading index an acceptable proxy for wing loading. Differences in relative wing loading index between sexes were tested using the two‐sample *t*‐test.

All statistical analyses were carried out in R (R Core Team 2024). Data are reported as mean ± standard deviation (SD). Visualisations were generated using the R package ggplot2 (Wickham 2016) and maps were created in ArcGIS Pro3.5.2 (ESRI 2025).

## 3 Results

### 3.1 Migration Patterns of Avocets

**FIGURE 1:**
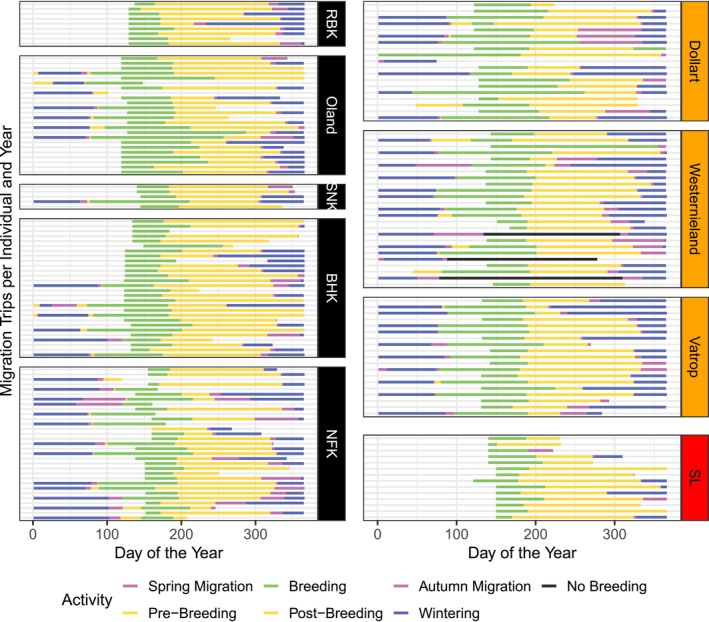
Phenology of the annual cycle per individual and year according to tagging location (black, German breeding colonies; orange, Dutch breeding colonies; red, Polish breeding colony; RBK, Rickelsbüller Koog; SNK, Sönke‐Nissen‐Koog; BHK, Beltringharder Koog; NFK, Neufelderkoog; SL, Szczecin Lagoon). Bars for each individual start with catching during breeding in the middle of the year; bar in the next line either continues the bar from the individual above (if > 1 year of data recorded) or starts again with breeding of another individual. Spring Migration = time spent on migration between departure from the wintering site and arrival at the Wadden Sea; Pre‐Breeding = period between arrival at the Wadden Sea and arrival at the breeding site; Breeding = time spent at the breeding site; Post‐Breeding = period between departure from the breeding site and departure from the Wadden Sea; Autumn Migration = time spent on migration between departure from the Wadden Sea and arrival at the wintering site; Wintering = time spent at the wintering site; No Breeding = time spent in the Wadden Sea without a breeding attempt in the given year.

On average, the tagged avocets departed from their breeding sites on 12 July ± 24.8 days, left the Wadden Sea on 5 November ± 41.3 days, and arrived at their wintering sites on 20 November ± 40.2 days (Table 3). On their return journey, avocets departed from the wintering sites on 17 March ± 14.8 days, reached the Wadden Sea on 26 March ± 19.0 days, and arrived at their breeding sites on 1 April ± 20.0 days on average (Table 3). Accordingly, the annual cycle of avocets could be divided into approximately three equal periods, with individuals spending on average 102 days at the breeding site, 116 days during the post‐breeding period, and 117 days at wintering sites. The remaining 30 days were divided among autumn and spring migration and the pre‐breeding period (Figure 1).

**TABLE 3:** Characteristics of migration parameters for avocets.

	Males				Females				All individuals			
	Mean	SD	Range	*n*	Mean	SD	Range	*n*	Mean	SD	Range	*n*
Autumn migration												
StopoverNo	1.3	1.3	0–4	42	2.6	2.7	0–13	40	2.0	2.6	0–13	115
MigrationDur [days]	10.8	20.0	0.5–131.7	49	18.7	23.1	0.6–79.3	43	14.8	21.2	0.3–131.7	130
MigrationSpeed [km day^−1^]	379.6	315.5	1–1143.5	49	349.5	312.9	20.1–1286.2	43	409.5	457.4	1–3127.1	128
DepBreeding	10 Jul	21.7	24 May–14 Oct	64	11 Jul	21.8	26 May–13 Sep	51	12 Jul	24.8	24 May–21 Dec	162
DepWadden	21 Nov	32.6	11 Jul–17 Feb	53	15 Oct	42.0	4 Aug–10 Jan	48	05 Nov	41.3	11 Jul–17 Feb	144
ArrWintering	04 Dec	28.2	19 Aug–19 Feb	50	05 Nov	44.3	07 Aug–13 Jan	43	20 Nov	40.2	07 Aug–19 Feb	131
Spring migration												
StopoverNo	1.7	1.5	0–5	22	2.8	2.6	1–9	18	2.2	2.3	0–10	57
MigrationDur [days]	4.7	5.7	0–19.1	25	14.3	18.4	0.7–61.8	18	4.4	16.1	0–71.3	59
MigrationSpeed [km day^−1^]	803.9	866.9	63.1–2821.6	21	506.6	684.3	25.4–3019.4	18	621.7	723.5	25.4–3019.4	52
DepWintering	19 Mar	11.0	4 Mar–12 Apr	24	17 Mar	17.0	26 Jan–11 Apr	18	17 Mar	14.8	26 Jan–25 Apr	58
ArrWadden	25 Mar	15.1	4 Mar–29 Apr	23	31 Mar	17.2	28 Feb–5 May	17	26 Mar	19.0	13 Feb–13 May	58
ArrBreeding	28 Mar	18.8	10 Mar–25 May	21	6 Apr	19.7	13 Mar–25 May	18	1 Apr	20.0	13 Feb–25 May	54
MigrationDist [km]	1276.7	629.7	137.3–2332.9	51	1928.2	978.5	818.9–5012.0	43	1502.6	995.9	137.3–5140.7	132
WinteringLat [°N]	45.2	5.3	36.5–53.3	52	39.8	8.0	13.4–49.4	43	43.2	8.4	10.5–53.7	136
WingLoading [g mm^−1^]	1.5	0.1	1.2–1.7	41	1.4	0.1	1.3–1.7	33	1.4	0.1	1.2–1.7	92

*Note:* Data represent mean, standard deviation (SD), range, and sample size (*n*). For statistical model output and abbreviations of the variables, see Table 2.

**FIGURE 2:**
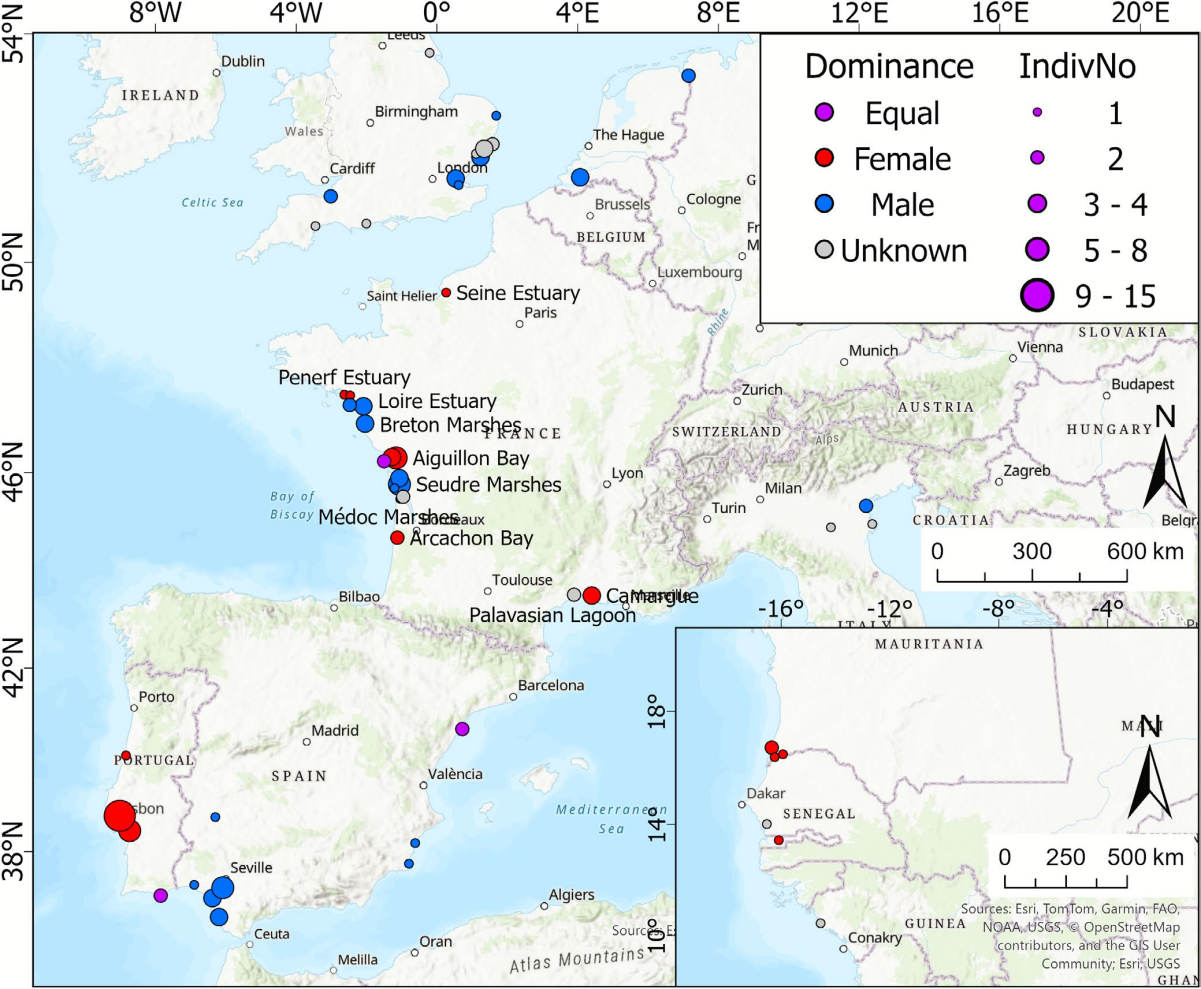
Wintering sites of avocets. Size of dot indicates number of tagged individuals wintering at the respective site; purple, sites used equally by females and males; red, sites used predominantly by females; blue, sites used predominantly by males; grey, sites used only by individuals of unknown sex. Names indicated for sites used by > 4 individuals and further sites mentioned in the text. See Figure A3 for detailed map of wintering sites on the French West coast.

**FIGURE 3:**
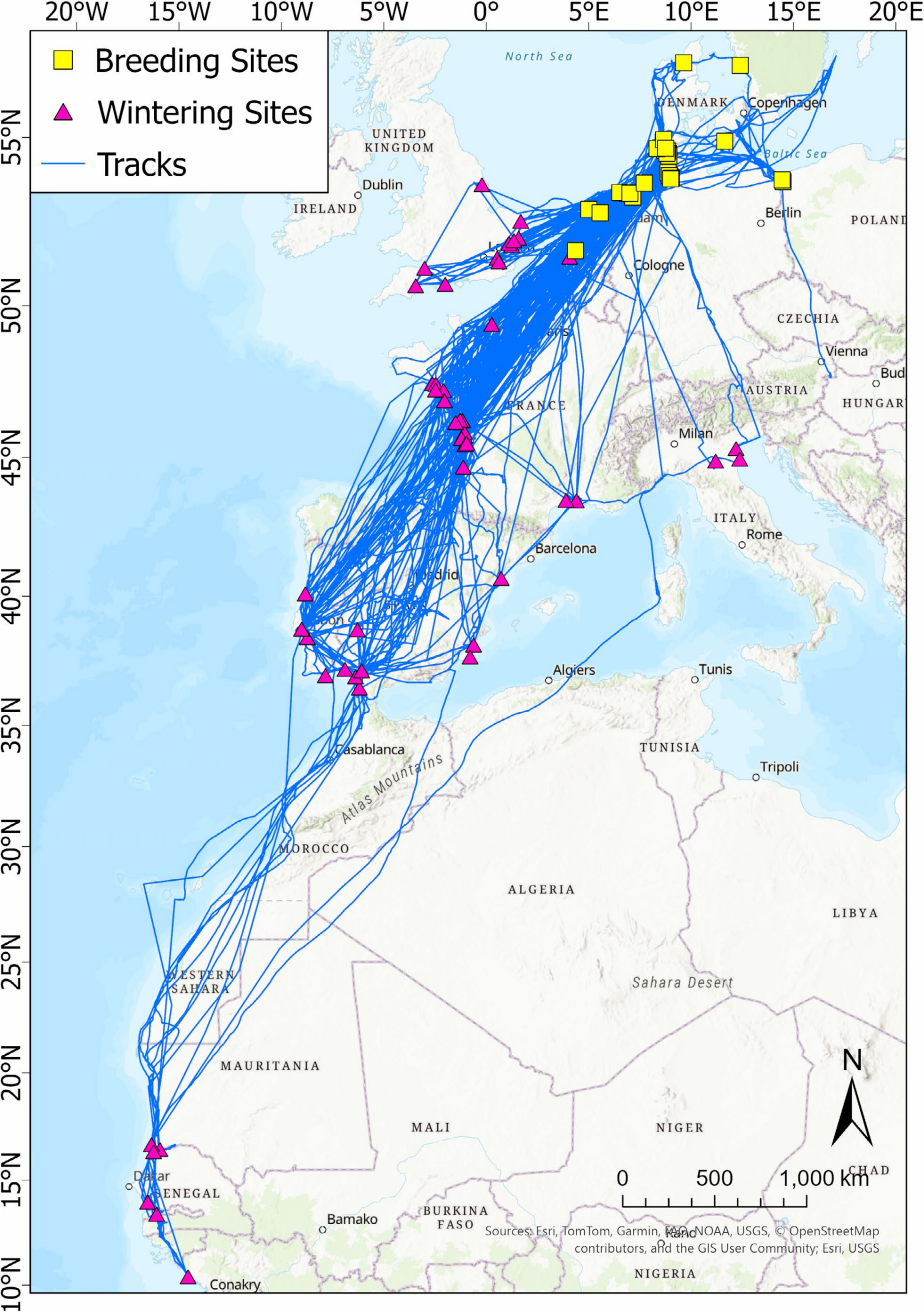
Migration tracks of all avocets in this study, showing breeding sites (yellow rectangles) and wintering sites (pink triangles). Several breeding and wintering sites were used by more than one individual. For comparison of migration tracks of females and males, and for avocets from Germany, The Netherlands and Poland see Figures A1 and A2.

Wintering sites ranged from the Humber Estuary in the United Kingdom (53°40′54″ N, 0°12′38″ W) in the north to the Kapatchez River in Guinea (10°26′47″ N, 14°33′30″ W) in the south (Table 3, Figures 2 and 3). The most frequently used wintering site was the Tejo Estuary in Portugal (38°47′46″ N, 9°01′04″ W), where 19 individuals spent at least one winter, followed by Aiguillon Bay (46°17′22″ N, 1°09′38″ W) and the Seudre Marshes (45°46′07″ N, 1°03′52″ W) in France, each used by eight individuals. The vast majority of tagged birds (87%) wintered in France, Portugal, the United Kingdom or Spain, following the EAF. Two females and two individuals of unknown sex proceeded migration along the EAF to Africa (Figures 2 and 3), and at least two of them spent multiple winters there. In contrast, two males remained in the Wadden Sea, wintering at Dollart (Netherlands/Germany; 53°17′32″ N, 7°09′33″ E). With the exception of one individual that migrated directly south from its breeding site, all avocets breeding at Szczecin Lagoon (Poland) moved to the Wadden Sea during the post‐breeding period before continuing southwards along routes similar to those of avocets breeding in the Wadden Sea colonies.

Five individuals made stopovers at the Mediterranean Sea, and three birds migrated directly from the Wadden Sea to their wintering sites in the Camargue, France (43°31′00″ N, 4°23′59″ E) or the Ebro Delta, Spain (40°41′53″ N, 0°43′39″ E). Additionally, one male and three individuals of unknown sex, including three of the 14 individuals breeding in Poland, followed a distinctly different migration route, crossing the Alps en route to their wintering sites in northern Italy (Figures 2 and 3).

Avocets made 2.0 ± 2.5 stopovers per migration trip and it took them 13.3 ± 19.8 days to travel 1502.6 ± 995.9 km, resulting in a mean migration speed of 470.8 ± 554.0 km day^−1^ (Table 3).

### 3.2 Effects of Sex, Season and Breeding Location on Migration

**FIGURE A1:**
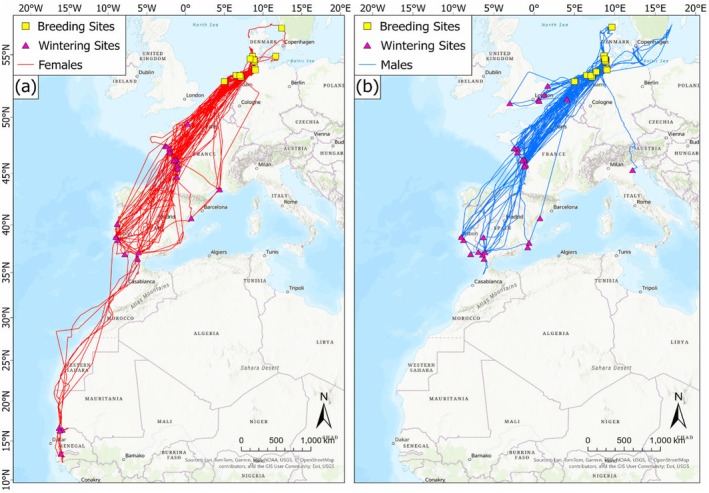
Migration tracks of (a) female and (b) male avocets, showing breeding sites (yellow rectangles) and wintering sites (pink triangles). Several breeding and wintering sites were used by more than one individual.

Females migrated significantly longer distances than males (*χ* ^2^ = 14.69, df = 1, *p* < 0.001; see Table 2 for detailed results of GLMMs and Table 3 for mean values ± SD, ranges, and sample sizes) and wintered at more southern latitudes (*χ* ^2^ = 5.51, df = 1, *p* = 0.019), indicating that females generally wintered further south (Figure 2, see also Figure A1 for migration tracks). There was no significant correlation between the departure date from the breeding site and migration distance (*ρ* = −0.005; *p* = 0.521; Spearman's rank correlation), and no sex‐specific difference in departure date from the breeding site (*χ* ^2^ = 0.14, df = 1, *p* = 0.707). However, females departed significantly earlier from the Wadden Sea (*χ* ^2^ = 12.3, df = 1, *p* < 0.001) and arrived significantly earlier at their wintering sites (*χ* ^2^ = 7.09, df = 1, *p* = 0.008) (Figure A4). Females also departed significantly earlier from their wintering sites in spring (*χ* ^2^ = 35.46, df = 1, *p* < 0.001), but there were no sex‐specific differences in arrival timing at either the Wadden Sea (*χ* ^2^ = 0.63, df = 1, *p* = 0.426) or the breeding sites (*χ* ^2^ = 1.97, df = 1, *p* = 0.161).

Migration duration was significantly longer in autumn than in spring (*χ* ^2^ = 9.34, df = 1, *p* = 0.002), whereas both the number of stopovers (*χ* ^2^ = 1541.6, df = 1, *p* < 0.001) and migration speed (*χ* ^2^ = 7.82, df = 1, *p* = 0.005) were significantly higher in spring (Figure A5).

Analysing the interactive effects of sex and season revealed a significantly longer migration duration in autumn for males (*p* = 0.013) but not for females (*p* = 0.110; Figure A6). Males also showed a significantly slower migration speed in autumn (*p* = 0.005), whereas females showed no seasonal variation in migration speed (*p* = 0.183). Furthermore, females tended towards a longer migration duration than males in both autumn (*p* = 0.109) and spring (*p* = 0.111), although these differences were slightly not significant (Table A3). There were no sex‐specific differences in migration speed during either autumn or spring migration (Table A4).

Females made significantly more stopovers in autumn (*p* = 0.013; GLMM), whereas no such sex‐specific difference was found in spring (*p* = 0.528; GLMM). Both sexes showed no seasonal variation in the number of stopovers (Table A2). This pattern was further supported by the significantly lower relative wing loading index of females compared with males (*t* = −2.24; *p* = 0.014; two‐sample *t*‐test).

**FIGURE A2:**
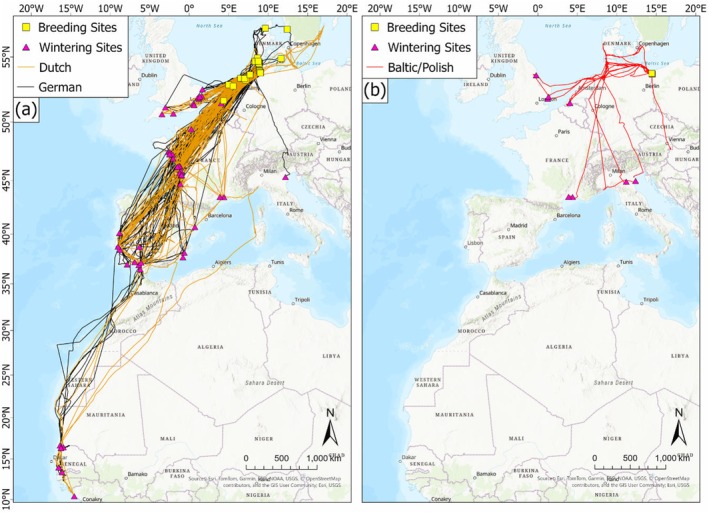
Migration tracks of avocets from (a) Dutch (orange) and German (black) breeding colonies, and from (b) a Baltic/Polish breeding colony, showing breeding sites (yellow rectangles) and wintering sites (pink triangles). Several breeding and wintering sites were used by more than one individual.

**FIGURE A4:**
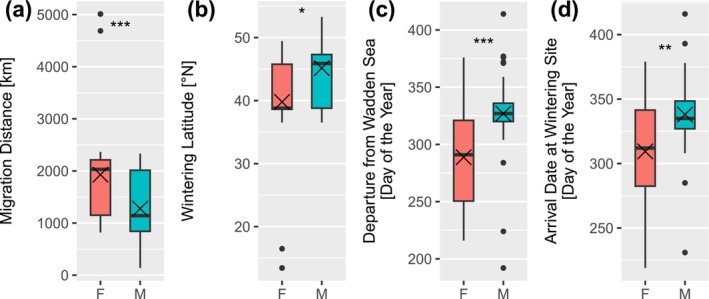
Autumn migration parameters with significant sex‐related differences (females (F), red; males (M), blue). (a) Migration distance (*n*_females_ = 43, *n*_males_ = 51). (b) Wintering latitude (*n*_females_ = 43, *n*_males_ = 52). (c) Date of departure from the Wadden Sea (*n*_females_ = 48, *n*_males_ = 53). (d) Date of arrival at the wintering site (*n*_females_ = 43, *n*_males_ = 50). Solid lines, medians; boxes, interquartile ranges; whiskers range up to 1.5 times the interquartile range; crosses, means; dots, outliers; Significance levels: n.s. not significant, *p < 0.05, **p < 0.01, ***p < 0.001.

**FIGURE A5:**
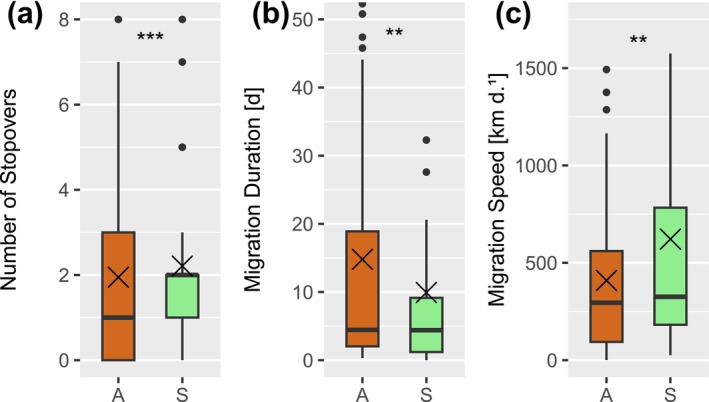
Seasonal differences in migration parameters (autumn (A), brown; spring (S), green). (a) Number of stopovers (*n*_autumn_ = 115; *n*_spring_ = 57). Outliers removed for clarity: Autumn (5 outliers; range: 10–13 stopovers); spring (3 outliers; 9–10 stopovers). (b) Migration duration (*n*_autumn_ = 130; *n*_spring_ = 59). Removed outliers: Autumn (9 outliers; 53.2–131.7 days); spring (4 outliers; 56.1–71.3 days). (c) Migration speed (*n*_autumn_ = 128; *n*_spring_ = 52). Removed outliers: Autumn (2 outliers; 2570.7–3127.1 km day^−1^); spring (5 outliers; 2091.2–3019.4 km day^−1^). For boxplot definitions and significance levels, see Figure A4.

**FIGURE A6:**
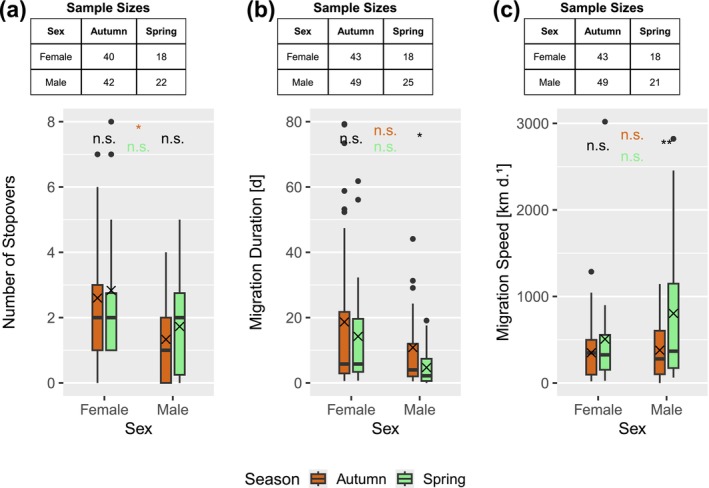
Differences in migration parameters with respect to season and sex. (a) Number of stopovers. Outliers removed for clarity: Female/autumn (2 outliers; range: 10–13 stopovers); female/spring (1 outlier; 9 stopovers). (b) Migration duration. Removed outlier: Male/autumn (1 outlier; 131.7 days). (c) Migration speed. Sample sizes indicated in tables above each graph. For boxplot definitions and significance levels, see Figure A4. Significance levels shown in black apply to intrasexual comparisons across seasons; significance levels applying to intersexual comparisons within seasons shown in brown (autumn) or green (spring).

Avocets breeding in the Wadden Sea (German and Dutch colonies) made significantly more stopovers (*χ* ^2^ = 5.9, df = 1, *p* = 0.015) and had a significantly longer migration duration (*χ* ^2^ = 6.08, df = 1, *p* = 0.014) than those breeding in the Baltic Sea region (Polish colony) (Table 4). The wintering latitude was also significantly lower for Wadden Sea breeders (*χ* ^2^ = 3.83, df = 1, *p* = 0.050), indicating that these birds overwintered further south than their Baltic conspecifics (see Figure A2 for migration tracks). Additionally, avocets from the Polish colony departed significantly later from the Wadden Sea (*χ* ^2^ = 4.11, df = 1, *p* = 0.043). There were no regional differences in terms of migration distance, departure from the breeding site, or arrival at the wintering site (Table 2).

**TABLE 4:** Characteristics of autumn migration parameters for avocets tagged in the Wadden Sea (Dutch (NL) and German (DE) breeding colonies) compared with individuals from a Polish colony (PL) in the Baltic Sea.

	Wadden Sea (DE, NL)				Baltic (PL)			
	Mean	SD	Range	*n*	Mean	SD	Range	*n*
StopoverNo	2.1	2.6	0–13	107	0.5	1.1	0–3	8
MigrationDur [days]	15.2	21.3	0.3–131.7	122	8.4	20.3	0.3–58.7	8
MigrationSpeed [km day^−1^]	356.0	328.0	1.0–1492.4	120	1212.4	1086.3	12.7–3127.1	8
WinteringLat [°N]	42.9	8.5	10.5–53.3	128	48.4	4.1	43.5–53.7	8
MigrationDist [km]	1537.5	1016.8	137.3–5140.7	124	961.1	192.2	744.1–1359.0	8
DepBreeding	13 Jul	25.4	24 May–21 Dec	148	7 Jul	16.8	31 May–30 Jul	14
DepWadden	3 Nov	40.7	11 Jul–17 Feb	135	2 Dec	43.1	29 Sep–17 Feb	9
ArrWintering	19 Nov	39.3	7 Aug–19 Feb	123	12 Dec	49.5	1 Oct–18 Feb	8

*Note:* Data represent mean, standard deviation (SD), range, and sample size (*n*). For statistical model output and abbreviations of the variables, see Table 2.

## 4 Discussion

### 4.1 Spatial and Temporal Migration Patterns

The avocets analysed in this study departed from their breeding sites on 12 July (on average), which was identical to the mean departure date reported by Eskildsen et al. (2025) for a smaller sample restricted to two of the nine colonies included in the current study. The onset of autumn migration from the Wadden Sea (5 November) was similar to that of a population migrating along the East Asian–Australasian Flyway (EAAF) (9 November; Wijethunge et al. 2024). While EAF avocets travelled longer distances (1502 km vs. 1142 km in EAAF), they arrived at the wintering sites at similar times (20 November vs. 22 November in EAAF; Wu et al. 2022). This similarity can only be explained by a shorter migration duration in the EAF population (13.3 days vs. 24 days in EAAF), resulting in a markedly higher migration speed (470.8 km day^−1^ vs. 58 km day^−1^ in EAAF; Wijethunge et al. 2024). Differences between the two flyways in spring were minimal, with EAAF birds leaving their wintering sites slightly later (22 March vs. 17 March) and reaching their breeding sites only a few days later (7 April vs. 1 April). These dates are also in line with earlier observations of avocets in Wadden Sea colonies, which arrived at their breeding sites between early March and early July (Hötker 2002).

In contrast, the closely related American avocet undertakes substantially longer migrations (2234.7 km), with longer durations (19.6 days), more stopovers (4.9 vs. 2 stopovers), and consequently arrives later at its breeding sites (10 May) (Clements et al. 2025). While American avocets are dependent on unpredictable ephemeral wetlands as stopover sites (Davis and Smith 2001; Ackerman et al. 2020; Clements et al. 2025), pied avocets can rely on a network of more predictable coastal wetlands (Li et al. 2024; Eskildsen et al. 2025), which likely allows a more energy‐efficient migration strategy and may explain differences in migratory variables between these two morphologically similar species on different continents (Pierce 1996). Nevertheless, both pied and American avocets appear to devote a considerable proportion of the migration time to stopovers relative to migration distance, suggesting that they follow an energy‐minimising rather than time‐minimising migration strategy (Clements et al. 2025; Eskildsen et al. 2025).

Compared with long‐distance migrating waders breeding in Siberia, such as grey plovers ( *Pluvialis squatarola* ; Catry et al. 2024) and Eurasian curlews (Pederson et al. 2022), avocets began their autumn migration later but initiated their spring migration earlier. The later onset of autumn migration can be explained by the much longer migration distances of grey plovers (6632–11,013 km) and curlews (3362 km) (Pederson et al. 2022; Catry et al. 2024). However, this explanation does not account for the earlier start of spring migration by avocets, which instead suggests that they spent proportionally more time at their breeding sites. Waders breeding in Siberia face the constraint of a short vegetation period, whereas avocets are less time‐limited in their breeding season (Wyndham 1986).

This interpretation is consistent with the annual cycle revealed in the present study, which showed similar durations of 102–117 days for the breeding, post‐breeding and wintering periods. Consequently, avocets spent far longer in the post‐breeding period than species such as Eurasian curlews, whose annual cycle is dominated by wintering (Pederson et al. 2022). Avocets along the EAAF, for instance, spent 195 days at their breeding sites (post‐breeding movements likely included) and 108 days at their wintering sites (Wijethunge et al. 2024), again pointing to a longer migration period compared with the EAF. Comparable pre‐ or post‐migratory movements have been reported in other species: lesser black‐backed gulls ( *Larus fuscus* ) showed spatial and temporal migration patterns similar to avocets but lacked such an extended post‐breeding period (Klaassen et al. 2012), whereas Mediterranean gulls displayed post‐migratory exploration during the pre‐breeding period, leading to longer spring than autumn migrations (Jankowiak et al. 2024). The prolonged post‐breeding period in the Wadden Sea close to the breeding sites thus appears to represent a relatively unique feature of the migration ecology of avocets breeding in the Wadden Sea.

Avocets of the EAF population are generally described as coastal migrants, with routes predominantly following western European coastlines and only rare inland movements (Glutz von Blotzheim et al. 1986). Our results largely support this view, in line with previous studies highlighting their strong preference for coastal over inland wetlands as stopover habitats along both the EAF and EAAF (Wu et al. 2022; Li et al. 2024; Wijethunge et al. 2024; Eskildsen et al. 2025). However, we also identified an alternative route linking Baltic Sea colonies—and to a lesser extent Wadden Sea colonies—with wintering sites in the Mediterranean, including trans‐Alpine crossings (see also Marchowski et al. 2023).

In contrast to Chambon et al. (2018), who reported, based on ringing data, that most avocets breeding in France were resident and only 15% migrated, we found that all tagged individuals showed at least some migratory behaviour, including short post‐breeding movements to the Dollart that may not be detectable by ringing alone. No birds overwintered at their breeding sites. Nevertheless, wintering‐site choice is known to be influenced by ambient temperature, with colder winters pushing individuals further south (Hötker 1998). Ongoing climate change may thus lead to shorter migrations and increasing residency (Visser et al. 2009; Lok et al. 2013), and avocets may increasingly remain at northern sites.

Despite these possible future shifts, most individuals (73%) in the current study wintered in France, Spain, or Portugal, with the Tejo Estuary emerging as the most frequently used site. This estuary is recognised as a crucial stepping stone along the EAF for many species; for example, it exhibits the highest network connectivity of black‐tailed godwit ( *Limosa limosa* ) sites, linking directly to more than 200 other localities (Beal et al. 2025). The importance of the Tejo Estuary for avocets may have further increased following declines at the nearby Sado Estuary, where habitat loss has reduced high‐tide roost availability by 20% (Belo et al. 2022). Taken together, these findings suggest that the strong wintering‐site fidelity of avocets may counteract any emerging trend towards shorter migration distances (Hötker 1998; Wu et al. 2022).

### 4.2 Sex‐Related Differences in Migration Patterns

Our results demonstrated that female avocets overwintered further south than their male conspecifics. The most northerly wintering sites, located in the Dutch Wadden Sea and the United Kingdom, were used exclusively by males. This finding aligns with earlier observations that females more frequently overwintered in Portugal, whereas males were more common in France during winter (Dietrich 1999).

The current results showed that females departed earlier from the Wadden Sea and consequently arrived earlier at the wintering sites, although Eskildsen et al. (2025) reported that females left the breeding sites later than males. Our data, however, indicated that the departure date from the breeding site was not correlated with migration distance. This suggests that males may spend a more extended post‐breeding period in the Wadden Sea, potentially prospecting for future breeding sites, as observed in other colonial species such as Mediterranean gulls (Jankowiak et al. 2024; Garthe et al. 2025). In contrast, in American avocets, both sexes show extended post‐breeding movements (Plissner et al. 2000). However, female pied avocets may prospect during migration or wintering, as indicated by the observation that two colour‐ringed females bred at former wintering sites (PM unpubl. data). Ausems et al. (2025) proposed that sex‐specific migratory routes could explain the earlier onset of migration in female Eurasian whimbrels ( *Numenius phaeopus* ) compared with successful males. However, our results do not support such a pattern, given that nearly all individuals followed the same migratory route.

The observed sex‐specific differences in migration timing are more likely to result from differing migration strategies. Along the EAF, females departed earlier from the wintering sites but tended to arrive later at the breeding sites, consistent with earlier observations by Hötker (2002). This can be explained by their generally longer migration duration and greater number of stopovers compared with males. The apparently contradictory finding of Eskildsen et al. (2025)—that males made longer stopovers during autumn migration—likely stems from their inclusion of stops made during the post‐breeding period, which the current study suggests are more prolonged in males.

Overall, these findings suggest a clear dichotomy in migration tactics between the sexes: males follow a ‘near and short’ migration (near distance, short duration), whereas females undertake a ‘far and long’ migration (far distance, long duration), ultimately resulting in similar migration speeds between the sexes. However, this apparent similarity may partly be an artefact of not including pre‐migratory fuelling in the calculation of migration speed (Lindström et al. 2019). The longer post‐breeding period of males (i.e., longer pre‐migratory fuelling) may compensate for the higher number of stopovers made by females during autumn migration. The longer migration distance of females (Vágási et al. 2016) may be associated with their lower relative wing loading index, probably indicative of superior flight efficiency (Norberg 1990). This may allow females to reach higher‐quality habitats located further away, thereby reducing competition with male conspecifics at wintering sites. A lower cold tolerance of the smaller‐bodied sex may also force females to winter further south (Ketterson and Nolan 1983). Along the EAAF, where no sex‐specific difference in migration distance has been detected, males depart from the wintering sites earlier (Wu et al. 2022). This suggests that males employ different migration strategies across the two flyways, both serving the same adaptive purpose of an earlier return to the breeding sites to maximise mating opportunities and secure high‐quality territories (Schmaljohann et al. 2022), given that delayed arrival is known to reduce breeding success (Hötker 1998).

In contrast, no such sex‐related (or seasonal) differences in migration timing have been reported for Eurasian curlews, as a representative Siberian‐breeding wader species (Pederson et al. 2022). For these long‐distance migrants, the short vegetation period at the breeding grounds and the longer migration distances likely constrain variability in migration timing (Wyndham 1986; Clements et al. 2025). In contrast, avocets breeding in the Wadden Sea are exposed to more flexible environmental conditions, allowing for greater temporal variability in their migration strategies (Eskildsen et al. 2025).

### 4.3 Differences Between Spring and Autumn Migration

The EAF population of avocets exhibited longer migration durations and lower migration speeds in autumn than in spring, as shown by Wijethunge et al. (2024) for the EAAF population of avocets. Most migratory birds travel faster in spring because of stronger time constraints (Klaassen et al. 2012; Duijns et al. 2019; Schmaljohann et al. 2022; Wright et al. 2022; Catry et al. 2024), given that their earlier arrival at the breeding site enhances reproductive success—particularly in males (Hötker 1998)—and may increase the likelihood of a second breeding attempt following nest failure (Gates et al. 2013).

In contrast to our initial prediction, avocets made more stopovers during spring migration along both flyways, suggesting that these spring stopovers must have been comparatively short. Avocets thus appear to perform a relatively leisurely autumn migration with fewer but longer stopovers, followed by a more hurried spring migration characterised by more frequent, brief stopovers. Zhao et al. (2017) likewise concluded that several Scolopacidae species migrating along the EAAF follow a time‐minimisation strategy during spring migration and an energy‐minimisation strategy during autumn migration. Longer stopovers in autumn would allow the birds to refuel more efficiently, improving body condition and enabling longer flight segments, whereas shorter stopovers in spring may result in poorer condition and thus shorter flight bouts requiring more frequent stops. This interpretation is supported by the finding that avocets spent more time at their main stopover site in autumn than in spring (Wu et al. 2022).

Ambient temperature is known to influence departure decisions in autumn and the selection of wintering sites (Glutz von Blotzheim et al. 1986; Hötker 1998), and other environmental factors are also likely to affect stopover strategies. For instance, dunlins ( *Calidris alpina* ) and bar‐tailed godwits ( *Limosa lapponica* ) tend to migrate with tailwinds in spring and face headwinds in autumn (Grönroos et al. 2012). Such conditions may explain the longer duration of autumn migration and the need for extended stopovers, given that flying in persistent headwinds is both energetically demanding and time‐consuming (Liechti and Bruderer 1998). A higher number of stopovers during spring migration may also help birds to assess the environmental conditions en route or at the breeding sites (Bauer et al. 2020), while during autumn migration, avocets may simply depart the Wadden Sea with high fuel loads once temperatures drop, flying directly or with few stops to their wintering areas. However, Schwemmer et al. (2021) noted that migration timing in Eurasian curlews was independent of weather conditions and was rather driven by genetic cues and resource availability at the breeding sites. Further research is therefore needed to clarify the relative importance of environmental and intrinsic factors shaping the pronounced seasonal differences in avocet migration patterns.

### 4.4 Differences in Migration Patterns Between Breeding Populations

Our results indicated that avocets breeding in the Wadden Sea wintered further south than those breeding in the Baltic Sea. This contrasts with Glutz von Blotzheim et al. (1986), who reported no difference in the wintering sites of Baltic and North Sea populations. However, the longer migration durations and higher number of stopovers observed for the Wadden Sea breeding population suggest some degree of regional variation. Although an extended migration duration could theoretically result from longer stopovers alone, it might be more plausibly explained by the greater migration distances travelled by the Wadden Sea birds. Nevertheless, the sample size of Polish avocets was relatively small compared with that of the Wadden Sea population and may not fully represent the entire range of migration patterns within the Baltic Sea population.

To enable comparison between both breeding populations within a unified dataset, we defined the onset of autumn migration as the time of departure from the Wadden Sea, and the initial flight of Polish individuals from their breeding colonies to the Wadden Sea was therefore not included in the calculations of migration distance, duration, and speed. Interestingly, Polish avocets tended to leave their breeding sites earlier than individuals breeding in the Wadden Sea but started autumn migration later, i.e., later departure from the Wadden Sea. This pattern suggests that they spent an extended period in the Wadden Sea, using it as a major staging and refuelling site prior to continuing their migration.

Although an alternative migratory route connects Baltic Sea colonies with Mediterranean wintering sites, running southwards and skirting the Alps to the east (Cramp and Simmons 1983), nearly all tracked Polish individuals moved westwards to the Wadden Sea before continuing south. Only a single bird, whose track was incomplete, appeared to follow this more direct route. Even the three individuals that eventually wintered in Italy initially travelled to the Wadden Sea before deviating from the common EAF route. The exceptional food availability and favourable foraging conditions in the Wadden Sea (Laursen et al. 2010) likely justify this apparent detour, making it a crucial hub even for Baltic breeders.

Hötker (1998) also reported differences in arrival times among avocets from various Wadden Sea colonies, suggesting that comparable temporal differences exist between the Wadden Sea and the Baltic Sea populations. However, our dataset did not include spring migration data for the Polish individuals and this theory thus remains speculative. Long‐term and more comprehensive tracking data are needed to clarify the temporal differences throughout the annual cycle between these regional breeding populations.

## 5 Conclusion

Our findings largely support the proposed predictions, highlighting the importance of the Wadden Sea region for the EAF avocet population and clearly demonstrating that males and females follow distinct migration strategies. (I) The comparatively short migration distances and delayed departures confirm that avocets adopt a time‐relaxed migration strategy, enabled by an extended post‐breeding period in the Wadden Sea. (II & III) Pronounced sex‐specific patterns emerged, with females undertaking longer migrations and wintering further south, whereas males remained longer near the breeding sites and returned earlier in spring, suggesting divergent strategies. (IV) Seasonal contrasts in migration, with longer durations and lower speeds in autumn versus faster but more fragmented travels in spring, reflect differing energetic and ecological constraints across seasons. (V) Geographical differences between the Wadden Sea and Baltic Sea populations highlight the central role of the Wadden Sea as a key hub linking northern breeding and southern wintering sites.

Overall, these results demonstrate that migration strategies in avocets are shaped by a combination of intrinsic (sex‐specific) and extrinsic (geographical and seasonal) factors, resulting in clear temporal differences between sexes and seasons, but also showing a high degree of individual variability. In light of declining population numbers in the Wadden Sea (Kleefstra et al. 2025), future research should explore how environmental variability and ongoing climate change may affect these strategies, particularly regarding potential shifts in migration distances and residency patterns within the EAF.

In addition, the pronounced post‐breeding movements observed towards the north and east suggest that avocets may engage in prospecting behaviour outside their main migratory corridor, potentially allowing them to identify and later colonise new breeding habitats. Such exploratory flights might explain how the recently established colony in the Szczecin Lagoon emerged on newly created artificial islands and may also be consistent with a possible Wadden Sea origin of this population. Understanding how migratory connectivity, exploratory movements, and habitat availability interact will be key to assessing future population trajectories along the EAF.

## Author Contributions

**Mads Eskildsen:** conceptualization (equal), data curation (equal), formal analysis (lead), investigation (equal), methodology (equal), project administration (equal), resources (equal), validation (equal), visualization (lead), writing – original draft (lead), writing – review and editing (lead). **Stefan Garthe:** conceptualization (equal), data curation (equal), funding acquisition (equal), investigation (supporting), methodology (equal), project administration (equal), resources (equal), supervision (supporting), writing – review and editing (equal). **Sjoerd Duijns:** data curation (equal), funding acquisition (equal), investigation (supporting), project administration (equal), resources (equal), writing – review and editing (equal). **Petra Manche:** data curation (equal), investigation (supporting), project administration (equal), resources (equal), writing – review and editing (equal). **Dominik Marchowski:** data curation (equal), funding acquisition (equal), investigation (supporting), project administration (equal), resources (equal), writing – review and editing (equal). **Łukasz Jankowiak:** data curation (equal), funding acquisition (equal), investigation (supporting), resources (equal), writing – review and editing (equal). **Marcin Sidelnik:** data curation (equal), funding acquisition (equal), investigation (supporting), resources (equal), writing – review and editing (equal). **Philipp Schwemmer:** conceptualization (equal), data curation (equal), formal analysis (supporting), funding acquisition (equal), investigation (equal), methodology (equal), project administration (equal), resources (equal), supervision (lead), validation (equal), visualization (supporting), writing – original draft (supporting), writing – review and editing (equal).

## Funding

Financial support for the acquisition of GPS loggers was provided in the context of the project ‘Unser Wattenmeervogel’ by the National Park Administration of Schleswig‐Holstein funded by the proWIN pro nature‐foundation. Parts of the study were funded by the German Federal Ministry of Research, Technology and Space (BMFTR) within the project TRICMA^2^ (grant no. FKZ 03 F0960 C), and by the German Federal Agency for Nature Conservation with funds from the German Federal Ministry for the Environment, Climate Action, Nature Conservation and Nuclear Safety (BMUKN) within the project TRACKBIRD (grant no. FKZ 3519861400). The Dutch part of this work was part of the program Wij & Wadvogels, with financial contributions from Waddenfonds, Vogelbescherming Nederland, Rijkswaterstaat, the Dutch Ministry of Agriculture, Nature and Food Quality, and the Dutch provinces Noord‐Holland, Groningen, and Friesland. Fieldwork at Szczecin Lagoon was conducted as part of the ongoing research activities of the Museum and Institute of Zoology, Polish Academy of Sciences, and co‐financed by the Polish Ministry of Science under the ‘Regional Excellence Initiative’ program (agreement no. RID/SP/0045/2024/01).

## Ethics Statement

The National Park Administration of Schleswig‐Holstein, Groninger Landschap, Staatsbosbeheer, and the Maritime Office in Szczecin gave permission to enter the breeding grounds of avocets. The treatment of birds complied with current EU laws. Permission for GPS logger deployment on avocets was given by the Ministry for Energy Transition, Climate Protection, Environment and Nature Schleswig‐Holstein (MEKUN SH, file number: V 242‐39334/2022 (41‐5/22)) and the Central Authority for Scientific Procedures on Animals in The Netherlands (permit number AVD25000202010465).

## Conflicts of Interest

The authors declare no conflicts of interest.

## Supporting information


Data S1:

## Figures and Tables

**FIGURE A3:**
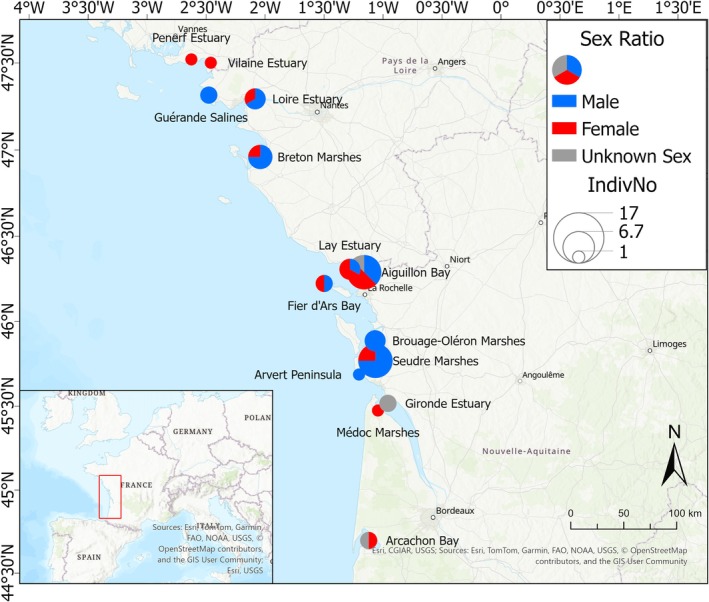
Wintering sites of avocets along the French west coast. Pie charts indicate number of tagged individuals wintering at the respective site. Colours depict proportions of males (blue), females (red), and avocets of unknown sex (grey) to number of tagged individuals.

## Data Availability

All the required data are uploaded as Supporting Information.

## Appendix A

**TABLE A1:** General information on tagged individuals by ascending ring ID.

Ring ID	Tagging date	Tagging place	Sex	Wing [mm]	Mass [g]	Rel. wing loading index [g mm^−1^]	Tarsus [mm]	Bill [mm]	Tag type	Catching method	Attachment method
3740153	12.05.2022	Vatrop	Female	236	350	1.48	91.3		OT‐9	Walk‐in trap	Leg‐loop harness
3740154	12.05.2022	Vatrop	Male	232	340	1.47	89.8	85	OT‐9	Walk‐in trap	Leg‐loop harness
3740156	12.05.2022	Vatrop	Female	225	357	1.59	87.1	81	OT‐9	Walk‐in trap	Leg‐loop harness
3783040	23.05.2023	Westernieland		232	343	1.48		87	OT‐9	Walk‐in trap	Leg‐loop harness
3783041	23.05.2023	Westernieland		238	315	1.32		87	OT‐9	Walk‐in trap	Leg‐loop harness
3783042	23.05.2023	Westernieland		233	302	1.30		75	OT‐9	Walk‐in trap	Leg‐loop harness
3785051	02.05.2022	Dollart	Male	236	334	1.42		91	OT‐9	Walk‐in trap	Leg‐loop harness
3785052	02.05.2022	Dollart	Female	234	319	1.36		84	OT‐9	Walk‐in trap	Leg‐loop harness
3785055	02.05.2022	Dollart	Female	231	374	1.62		80	OT‐9	Walk‐in trap	Leg‐loop harness
3785056	02.05.2022	Dollart	Male	231	346	1.50		86	OT‐9	Walk‐in trap	Leg‐loop harness
3785057	17.05.2022	Westernieland	Male	229	332	1.45	98	86	OT‐9	Walk‐in trap	Leg‐loop harness
3785058	17.05.2022	Westernieland	Male	234	373	1.60	91.6	85	OT‐9	Walk‐in trap	Leg‐loop harness
3785059	17.05.2022	Westernieland	Female	227	332	1.46	80.5	78	OT‐9	Walk‐in trap	Leg‐loop harness
3785062	31.05.2022	Westernieland	Female	226	312	1.38	76.8	74	OT‐9	Walk‐in trap	Leg‐loop harness
3785074	08.05.2023	Dollart		234	331	1.41		80	OT‐9	Walk‐in trap	Leg‐loop harness
3785075	08.05.2023	Dollart		218	298	1.37		78	OT‐9	Walk‐in trap	Leg‐loop harness
3785076	08.05.2023	Dollart		235	336	1.43		86	OT‐9	Walk‐in trap	Leg‐loop harness
3785077	08.05.2023	Dollart		235	306	1.30		84	OT‐9	Walk‐in trap	Leg‐loop harness
3785078	08.05.2023	Dollart		231	287	1.24		70	OT‐9	Walk‐in trap	Leg‐loop harness
3785079	22.05.2023	Vatrop		233	313	1.34		86	OT‐9	Walk‐in trap	Leg‐loop harness
3785080	22.05.2023	Vatrop		234	375	1.60		84	OT‐9	Walk‐in trap	Leg‐loop harness
3785083	16.06.2023	Westernieland		228	303	1.33		77	OT‐9	Walk‐in trap	Leg‐loop harness
3785206	18.05.2021	Westernieland		232	375	1.62	88.4	89	OT‐9	Walk‐in trap	Leg‐loop harness
3785208	18.05.2021	Westernieland		225	338	1.50	84	79	OT‐9	Walk‐in trap	Leg‐loop harness
3785213	26.05.2021	Westernieland		224	287	1.28	77.8	79	OT‐9	Walk‐in trap	Leg‐loop harness
3787364	11.05.2023	Vatrop		226	333	1.47	78	76	OT‐9	Walk‐in trap	Leg‐loop harness
3787365	11.05.2023	Vatrop		239	355	1.49	91.8	92	OT‐9	Walk‐in trap	Leg‐loop harness
3787366	11.05.2023	Vatrop		226	308	1.36	88	83	OT‐9	Walk‐in trap	Leg‐loop harness
3787367	11.05.2023	Vatrop		238	316	1.33	79.5	84	OT‐9	Walk‐in trap	Leg‐loop harness
5378103	13.05.2024	Beltringharder Koog	Female	224	309	1.38	80.9	76.3	OT‐6	Walk‐in trap	Body harness
5378104	13.05.2024	Beltringharder Koog	Female	231	307	1.33	85.0	80.6	OT‐6	Walk‐in trap	Body harness
5378105	13.05.2024	Beltringharder Koog	Male	230	343	1.49	88.8	82.0	OT‐6	Walk‐in trap	Body harness
5378106	13.05.2024	Beltringharder Koog	Male	232	366	1.58	83.2	88.5	OT‐6	Walk‐in trap	Body harness
5378107	13.05.2024	Beltringharder Koog	Male	225	314	1.40	82.3	78.9	OT‐6	Walk‐in trap	Body harness
5378128	16.05.2024	Rickelsbüller Koog	Male	228			87.6	85.7	OT‐6	Walk‐in trap	Body harness
5378132	28.05.2024	Beltringharder Koog	Female	212	302	1.42	78.6	80.0	OT‐6	Walk‐in trap	Body harness
5423722	03.06.2020	Neufelderkoog	Male	225	353	1.57	82.8		OT‐9	Walk‐in trap	Body harness
5423723	03.06.2020	Neufelderkoog	Male	235	305	1.30	77.7		OT‐9	Walk‐in trap	Body harness
5423724	03.06.2020	Neufelderkoog	Male	224	293	1.31	87.2		OT‐9	Walk‐in trap	Body harness
5423742	18.05.2022	Neufelderkoog	Female	217	313	1.44	80.1	80.0	OT‐9	Walk‐in trap	Body harness
5423743	18.05.2022	Neufelderkoog	Male	234	379	1.62	90.8	88.0	OT‐9	Walk‐in trap	Body harness
5423748	20.05.2022	Sönke‐Nissen‐Koog	Female	225	319	1.42	87.2	77.0	OT‐9	Walk‐in trap	Body harness
5423749	20.05.2022	Sönke‐Nissen‐Koog	Male	235	390	1.66	93.6	88.0	OT‐9	Walk‐in trap	Body harness
5424769	25.05.2021	Sönke‐Nissen‐Koog	Male	230	353	1.53	84.9	87.0	OT‐9	Walk‐in trap	Body harness
5424770	25.05.2021	Sönke‐Nissen‐Koog	Male	232	373	1.61	89.8	98.0	OT‐9	Walk‐in trap	Body harness
5424794	09.06.2021	Neufelderkoog	Female	227	285	1.26	82.9		OT‐9	Walk‐in trap	Body harness
5424795	09.06.2021	Neufelderkoog	Female	229	323	1.41	88.9		OT‐9	Walk‐in trap	Body harness
5424796	09.06.2021	Neufelderkoog	Female	220	318	1.45	82.8		OT‐9	Walk‐in trap	Body harness
5424797	09.06.2021	Neufelderkoog	Male	239	372	1.56	88.2		OT‐9	Walk‐in trap	Body harness
5424798	18.05.2022	Neufelderkoog	Male	228	339	1.49	86.9		OT‐9	Walk‐in trap	Body harness
5424800	18.05.2022	Neufelderkoog	Female	230	322	1.40	77.5	78.0	OT‐9	Walk‐in trap	Body harness
5437056	31.05.2022	Neufelderkoog	Female	230	322	1.40	85.3	79.0	OT‐9	Walk‐in trap	Body harness
5437057	31.05.2022	Neufelderkoog	Male	234	347	1.48	88.3	90.0	OT‐9	Walk‐in trap	Body harness
5437058	31.05.2022	Neufelderkoog	Male	225	276	1.23	83.8	81.0	OT‐9	Walk‐in trap	Body harness
5437059	31.05.2022	Neufelderkoog	Female	220	298	1.35	79.7	79.0	OT‐9	Walk‐in trap	Body harness
5437073	01.06.2023	Neufelderkoog	Male	240	344	1.43	87.4	89.0	OT‐9	Walk‐in trap	Body harness
5437074	01.06.2023	Neufelderkoog	Female	224	302	1.35	79.9	75.0	OT‐9	Walk‐in trap	Body harness
5437075	01.06.2023	Neufelderkoog	Male	235	331	1.41	86.4	83.0	OT‐9	Walk‐in trap	Body harness
5437078	28.04.2024	Oland	Female	220	338	1.54	84.8	77.4	OT‐6	Walk‐in trap	Body harness
5437079	03.05.2024	Beltringharder Koog	Female	226	345	1.53	84.4	78.0	OT‐6	Walk‐in trap	Body harness
5437080	03.05.2024	Beltringharder Koog	Female	219	322	1.47	82.8	83.5	OT‐6	Walk‐in trap	Body harness
5437081	03.05.2024	Beltringharder Koog	Male	230	306	1.33	79.7	80.4	OT‐6	Walk‐in trap	Body harness
5437083	03.05.2024	Beltringharder Koog	Female	230	326	1.42	88.4	85.0	OT‐6	Walk‐in trap	Body harness
5437084	03.05.2024	Beltringharder Koog	Female	226	295	1.31	79.5	80.0	OT‐6	Walk‐in trap	Body harness
5437085	03.05.2024	Beltringharder Koog	Male	225	388	1.72	88.8	83	OT‐6	Walk‐in trap	Body harness
5437089	08.05.2024	Rickelsbüller Koog	Male	229	365	1.59	84.8	88.0	OT‐6	Walk‐in trap	Body harness
5437090	08.05.2024	Rickelsbüller Koog	Male	224	360	1.61	94.8	90.0	OT‐6	Walk‐in trap	Body harness
5437091	08.05.2024	Rickelsbüller Koog	Female		336		81.2	81.6	OT‐6	Walk‐in trap	Body harness
5437092	08.05.2024	Rickelsbüller Koog	Female		326		83.9	78.9	OT‐6	Walk‐in trap	Body harness
5437093	08.05.2024	Rickelsbüller Koog	Female		358		87.4	88.0	OT‐6	Walk‐in trap	Body harness
5437095	08.05.2024	Rickelsbüller Koog	Male		338		91.2	86.1	OT‐6	Walk‐in trap	Body harness
5437096	08.05.2024	Rickelsbüller Koog	Male				87.3	85.1	OT‐6	Walk‐in trap	Body harness
5437097	08.05.2024	Rickelsbüller Koog	Male		320		82.5	77.5	OT‐6	Walk‐in trap	Body harness
5442609	29.04.2023	Oland	Male	235	360	1.53	83.0	90.0	OT‐9	Walk‐in trap	Body harness
5442610	29.04.2023	Oland	Male	236	375	1.59	91.4	85.0	OT‐9	Walk‐in trap	Body harness
5442611	29.04.2023	Oland	Male	234	329	1.41	87.7	89.0	OT‐9	Walk‐in trap	Body harness
5442612	29.04.2023	Oland	Male	234	325	1.39	86.4	89.0	OT‐9	Walk‐in trap	Body harness
5442613	29.04.2023	Oland	Female	220	310	1.41	78.5	75.0	OT‐9	Walk‐in trap	Body harness
5442614	03.05.2023	Beltringharder Koog	Female	232	312	1.34	75.9	75.0	OT‐9	Walk‐in trap	Body harness
5442615	03.05.2023	Beltringharder Koog	Female	223	315	1.41	79.9	76.0	OT‐9	Walk‐in trap	Body harness
5442616	03.05.2023	Beltringharder Koog	Male	225	349	1.55	79.7	91.0	OT‐9	Walk‐in trap	Body harness
5442617	03.05.2023	Beltringharder Koog	Female	224	344	1.54	79.0	84.0	OT‐9	Walk‐in trap	Body harness
5442618	03.05.2023	Beltringharder Koog	Male	227	320	1.41	83.3	82.0	OT‐9	Walk‐in trap	Body harness
5442619	03.05.2023	Beltringharder Koog	Male	233	344	1.48	88.8	90.0	OT‐9	Walk‐in trap	Body harness
5442620	07.05.2023	Oland	Male	237	340	1.43	88.3	84.0	OT‐9	Walk‐in trap	Body harness
5442621	07.05.2023	Oland	Male	229	342	1.49	84.5	83.0	OT‐9	Walk‐in trap	Body harness
5442622	07.05.2023	Oland	Male	235	356	1.51	85.7	87.0	OT‐9	Walk‐in trap	Body harness
5442623	07.05.2023	Oland	Male	232	364	1.57	92.1	88.0	OT‐9	Walk‐in trap	Body harness
5442627	12.05.2023	Beltringharder Koog	Male	238	363	1.53	83.1	89.0	OT‐9	Walk‐in trap	Body harness
5442628	12.05.2023	Beltringharder Koog	Female	227	292	1.29	82.8	80.0	OT‐9	Walk‐in trap	Body harness
5442629	12.05.2023	Beltringharder Koog	Female	231	346	1.50	85.6	78.0	OT‐9	Walk‐in trap	Body harness
5442630	12.05.2023	Beltringharder Koog	Male	233	387	1.66	86.0	89.0	OT‐9	Walk‐in trap	Body harness
5442644	28.04.2024	Oland	Female	223	354	1.59	87.1	81.3	OT‐6	Walk‐in trap	Body harness
5442645	28.04.2024	Oland	Female	224	387	1.73	87.6	79.3	OT‐6	Walk‐in trap	Body harness
5442646	28.04.2024	Oland	Male	225	348	1.55	82.7	79.1	OT‐6	Walk‐in trap	Body harness
5442647	28.04.2024	Oland	Female	232	332	1.43	82.6	78.9	OT‐6	Walk‐in trap	Body harness
5442648	28.04.2024	Oland	Male	224	304	1.36	86.4	85.1	OT‐6	Walk‐in trap	Body harness
5442649	28.04.2024	Oland	Female	236	332	1.41	83.8	78.1	OT‐6	Walk‐in trap	Body harness
5442650	28.04.2024	Oland	Male	234	319	1.36	89.4	88.8	OT‐6	Walk‐in trap	Body harness
FS16648	20.05.2023	Szczecin Lagoon		228			91.9	89.6	INTERREX 18	Walk‐in trap	Body harness
FS16649	20.05.2023	Szczecin Lagoon		222			97.5	80.6	INTERREX 18	Walk‐in trap	Body harness
FS16650	20.05.2023	Szczecin Lagoon		232			84.8	85.7	INTERREX 18	Walk‐in trap	Body harness
FS60401	20.05.2023	Szczecin Lagoon		229			91.6	79.6	INTERREX 18	Walk‐in trap	Body harness
FS60402	20.05.2023	Szczecin Lagoon		228			84.0	77.5	INTERREX 18	Walk‐in trap	Body harness
FS60424	29.05.2024	Szczecin Lagoon							INTERREX 18	Walk‐in trap	Body harness
FS60425	29.05.2024	Szczecin Lagoon			300				INTERREX 18	Walk‐in trap	Body harness
FS60426	29.05.2024	Szczecin Lagoon			325				INTERREX 18	Walk‐in trap	Body harness
FS60427	29.05.2024	Szczecin Lagoon			315				INTERREX 18	Walk‐in trap	Body harness
FS60428	29.05.2024	Szczecin Lagoon			305				INTERREX 18	Walk‐in trap	Body harness
FS60429	29.05.2024	Szczecin Lagoon			305				INTERREX 18	Walk‐in trap	Body harness
FS60430	29.05.2024	Szczecin Lagoon			295				INTERREX 18	Walk‐in trap	Body harness
FS60431	29.05.2024	Szczecin Lagoon			285				INTERREX 18	Walk‐in trap	Body harness
FS60432	29.05.2024	Szczecin Lagoon			320				INTERREX 18	Walk‐in trap	Body harness

*Note:* Ring IDs shown in same colour indicate a breeding pair where both partners were tagged (e.g., 5378103 and 5378106).

**TABLE A2:** Results of generalised linear mixed model analysing combined effect of sex and season on number of stopovers.

	Estimate	Std. error	*z*‐value	*p*	
(Intercept)	0.73	0.17	4.40	< 0.001	***
Male	−0.59	0.24	−2.47	0.013	*
Spring	−0.09	0.18	−0.52	0.602.	n.s.
Male:Spring	0.41	0.29	1.41	0.159	n.s.

*Note:* Significance levels: **p* < 0.05, ***p* < 0.01, ****p* < 0.001.

**TABLE A3:** Results of the generalised linear mixed model analysing the combined effect of sex and season on migration duration.

	Estimate	Std. error	*z*‐value	*p*	
(Intercept)	2.59	0.22	11.64	< 0.001	***
Male	−0.48	0.30	−1.60	0.109	n.s.
Spring	−0.51	0.32	−1.60	0.110	n.s.
Male:Spring	−0.19	0.42	−0.45	0.651	n.s.

*Note:* For significance levels see Table A2.

**TABLE A4:** Results of generalised linear mixed model analysing combined effect of sex and season on migration speed.

	Estimate	Std. error	*z*‐value	*p*	
(Intercept)	5.84	0.16	37.13	< 0.001	***
Male	0.06	0.22	0.26	0.792	n.s.
Spring	0.38	0.28	1.33	0.183	n.s.
Male:Spring	0.34	0.38	0.88	0.378	n.s.

*Note:* For significance levels see Table A2.
